# Whole-rock and mineral chemical data from a profile of the ~900 Ma Niutishan Fe-Ti-rich sill in XuZhou, North China

**DOI:** 10.1016/j.dib.2018.10.013

**Published:** 2018-10-09

**Authors:** Xiangdong Su, Peng Peng, Chong Wang, Fengbo Sun, Zhiyue Zhang, Xiaotong Zhou

**Affiliations:** a State Key Laboratory of Lithospheric Evolution, Institute of Geology and Geophysics, Chinese Academy of Sciences, P.O. Box 9825, Beijing 100029, China; b University of Chinese Academy of Sciences, Beijing 100049, China

## Abstract

In this article, the geochemical data of a ~900 Ma mafic sill from Xuzhou, North China are reported. These datasets include 37 whole-rock major and trace element data, and 21 major element data on plagioclase, 20 major element data on clinopyroxene crystals, 10 major element data on apatite crystals, 7 major element data of preserved coexisting titano-magnetite and ilmenite grains, 36 major element data on different types of ilmenite crystals, 13 major element and 11 trace element data on titanite. These data are associated with Su et al. “Petrogenesis of a ~900 Ma mafic sill from Xuzhou, North China: Implications for the genesis of Fe-Ti-rich rocks” (Su et al., 2018), and in which their characters and variations were interpreted and discussed.

**Specifications table**

**Table:** 

Subject area	*Geology*
More specific subject area	*Petrology*
Type of data	*Table*
How data was acquired	*X-ray fluorescence spectroscopy (AXIOS Minerals); Inductively Coupled Plasma Mass Spectrometry (ELEMENT); Electron probe microanalysis (JEOL JXA 8100 and CAMECA SX Five FE); LA-ICP-MS (Agilent 7500a ICP-MS coupled with a Resonetic RESOLution M–50 ArF–Excimer laser source)*
Data format	*Analyzed*
Experimental factors	*The erosion surface of sample was carefully removed and fresh samples were made for sample powders and thin sections*
Experimental features	*For major elements, sample powders, ~0.5* *g for each, were fused into a glass bead with ~5.0* *g of lithium tetraborate; whole-rock trace element analyses were determined using an ELEMENT instrument after HNO*_*3*_*+ HF digestion of approximately 40* *mg of sample powder in a Teflon vessel; thin and polished sections of the samples that were coated with carbon were made for the analysis of major elements on different minerals.*
Data source location	*Niutishan village (N 34°07׳03.87", E 117°40׳30.48"), Xuzhou city, China*
Data accessibility	*Data is with this article*
Related research article	*Xiangdong Su, Peng Peng, Chong Wang, Fengbo Sun, Zhiyue Zhang, Xiaotong Zhou, Petrogenesis of a ~900* *Ma mafic sill from Xuzhou, North China: Implications for the genesis of Fe-Ti-rich rocks. Lithos (2018)**[1]*.

**Value of the data**

• The whole-rock and mineral chemical data from a specific profile of the Niutishan mafic sill, one of the ~900 Ma sills from Xuzhou, North China were firstly reported.
• The datasets give basic information for a representative Fe-Ti-rich component-bearing mafic sill, and can be used for further investigation.
• This Fe-Ti-rich sill is characterized by the presence of >5 vol% of titanite, and thus compositions of this mineral are of broad interesting for the related community.

## 1 Data

In this paper, we report whole-rock and minerals geochemical data from a specific profile of a ~900 Ma mafic sill in XuZhou, North China. These datasets include 37 whole-rock major and trace element data (Table S1), and 21 major element data on plagioclase (Table 1), 20 major element data on clinopyroxene crystals (Table 2), 10 major element data on apatite crystals (Table 3), 7 major element data of preserved coexisting titano-magnetite and ilmenite grains (Table 4), 36 major element data on different types of ilmenite crystals (Table 5), 13 major element (Table 6) and 11 trace element data on titanite (Table 7). These data are associated with Su et al. “Petrogenesis of a ~900 Ma mafic sill from Xuzhou, North China: Implications for the genesis of Fe-Ti-rich rocks” [1]. The variation of the listed data (both of the whole-rock and minerals chemical compositions) from the specific profile of the sill are significant. The highest values for MgO, Cr and the most primitive plagioclase composition occur in *LZa* (-19.4 to -21.8 m), the highest values for FeO_t_ and TiO_2_ occur in *LZb* (-17.7 to -18.7 m), the highest value for P_2_O_5_ occurs in *LZc* (-16.7 to -17.7 m), and the most enriched incompatible elements occur in the *MZ* (-3.6 to -16.7). The uppermost rocks show the near mirror image of the lower section [1].

**Table 1:** Representative microprobe analyses of plagioclase from the Niutishan mafic sill (in wt.%).

**Sample point**	**Major zone**	**Height (m)**	**SiO**_**2**_	**TiO**_**2**_	**Al**_**2**_**O**_**3**_	**FeO**	**MgO**	**CaO**	**Na**_**2**_**O**	**K**_**2**_**O**	**Total**	**An**
1-pl-m1	*CM*	-0.02	52.781	0.111	28.408	1.159	0.513	12.516	4.245	0.155	99.93	61.4
3-pl-1		-0.5	53.159	0.099	28.604	0.753	0.084	8.618	4.199	0.248	99.92	61.7
8-pl-2	*UZ*	-2.6	55.947	0.069	27.782	0.58	0.072	10.359	5.371	0.262	100.46	50.8
13-pl-1	*MZ*	-5.02	64.114	0.006	20.626	0.804	0.017	3.704	10.15	0.086	99.53	16.7
13-pl-2			67.767	0.027	19.63	0.277	0.05	0.543	11.229	0.091	99.63	2.6
13-pl-3			67.519	0	19.522	0.465	0.036	0.313	11.409	0.07	99.33	1.5
15-pl-4		-6.0	67.01	0.007	20.01	0.572	0.047	1.229	11.009	0.071	99.96	5.8
15-pl-5			67.552	0.012	20.321	0.926	0.109	0.906	10.851	0.133	100.83	4.4
18-pl-1		-7.5	55.188	0.094	26.802	0.468	0.049	9.931	5.615	0.309	98.46	48.5
20-pl-2		-8.4	61.153	0.046	24.379	0.298	0.01	6.388	7.482	0.462	100.24	31.2
22-pl-4		-10.8	57.664	0.035	26.136	0.389	0.012	8.862	6.205	0.28	99.59	43.4
23-pl-1		-11.8	57.293	0.037	26.861	0.394	0.029	9.545	5.872	0.277	100.31	46.6
29-pl-1		-16.7	56.435	0.028	27.572	0.504	0.035	10.02	5.795	0.362	100.76	47.9
32-pl-4	*LZb*	-17.7	55.558	0.026	27.288	0.531	0.059	10.344	5.45	0.251	99.51	50.4
33-pl-1		-18.2	61.607	0	23.229	0.286	0.008	5.157	7.855	0.678	98.82	25.6
34-pl-2		-18.7	59.411	0.004	24.94	0.311	0.017	6.97	7.181	0.377	99.21	34.1
35-pl-2	*LZa*	-19.4	52.587	0.077	27.763	0.775	0.114	13.394	3.94	0.318	98.97	64.1
36-pl-2		-19.9	53.19	0.076	28.29	0.741	0.107	12.566	4.224	0.324	99.52	61.0
37-pl-1		-20.4	52.595	0.057	28.086	0.724	0.11	12.851	4.315	0.195	98.94	61.5
38-pl-1		-21.4	53.843	0.073	27.309	0.64	0.091	11.443	4.968	0.186	98.56	55.4
40-pl-1		-21.8	53.058	0.093	28.06	0.727	0.112	12.95	4.172	0.17	99.37	62.6

An= 100[Ca/(Ca + Na +K)].

**Table 2:** Representative microprobe analyses of clinopyroxene from the Niutishan mafic sill (in wt%).

**Sample point**	**Major zone**	**Height (m)**	**SiO**_**2**_	**TiO**_**2**_	**Al**_**2**_**O**_**3**_	**Cr**_**2**_**O**_**3**_	**FeO**	**MgO**	**CaO**	**Na**_**2**_**O**	**NiO**	**Total**	**Mg-no.**	**En**
1-cpx-p1	*CM*	-0.02	50.436	1.063	2.893	0.281	10.178	16.304	17.935	0.265	0.016	99.37	0.74	46.7
3-cpx-2		-0.5	50.952	1.05	3.823	0.213	9.905	15.458	18.733	0.366	0	100.5	0.74	44.8
6-cpx-1	*UZ*	-1.6	50.504	1.067	2.245	0.027	10.917	12.506	22.293	0.295	0.001	99.86	0.67	36.1
8-cpx-1		-2.6	50.759	1.139	2.778	0.052	11.5	14.313	19.179	0.307	0	100.03	0.69	41.4
10-cpx-3	*MZ*	-3.6	50.286	1.049	2.118	0	13.774	12.91	19.478	0.232	0.001	99.85	0.63	37.3
15-cpx-4		-6.0	50.665	0.873	1.545	0.008	15.989	10.525	20.246	0.237	0.003	100.09	0.54	30.9
18-cpx-1		-7.5	49.343	0.872	1.785	0	15.468	10.297	20.728	0.187	0.004	98.68	0.54	30.4
20-cpx-1		-8.4	50.161	0.733	1.436	0	16.508	9.915	20.571	0.221	0.01	99.56	0.52	29.2
22-cpx-3		-10.9	50.723	0.524	0.755	0	23.311	6.616	18.043	0.22	0	100.19	0.34	20.3
23-cpx-2		-11.8	50.283	0.812	1.25	0	15.006	10.676	21.038	0.217	0	99.28	0.56	31.2
24-cpx-1		-13.3	49.941	0.4	0.78	0.012	24.245	5.709	19.563	0.204	0	100.85	0.30	17.1
29-cpx-1		-16.7	50.34	0.37	0.75	0.02	20.00	6.34	21.89	0.14	0.00	99.85	0.36	19.0
32-cpx-1	*LZb*	-17.7	51.46	0.91	1.78	0.00	13.07	12.72	20.35	0.25	0.01	100.56	0.63	36.7
33-cpx-2		-18.2	49.63	1.28	2.88	0.00	12.72	12.69	19.90	0.25	0.00	99.36	0.64	37.2
34-cpx-1		-18.7	50.42	0.95	2.07	0.02	11.49	13.36	20.52	0.29	0.01	99.12	0.67	38.7
36-cpx-1	*LZa*	-19.9	51.453	0.798	2.169	0.03	10.663	15.362	19.075	0.275	0	99.83	0.72	43.8
37-cpx-1		-20.4	50.25	0.949	2.196	0.033	11.357	14.942	18.832	0.229	0.047	98.84	0.70	42.9
38-cpx-1		-20.9	51.776	0.95	2.395	0.016	10.527	15.388	19.191	0.292	0.003	100.54	0.72	43.9
39-cpx-1		-21.4	51.405	0.938	1.73	0	13.252	14.214	17.762	0.283	0	99.58	0.67	41.3
40-cpx-2		-21.8	51.099	0.859	2.178	0.08	10.137	15.537	19.188	0.282	0.021	99.38	0.73	44.4

Mg-no.= Mg /(Mg + Fe^2+^); En= 100 [Mg/(Mg + Ca + Fe^2+^)]. Fe^2+^ is calculated from stoichiometry.

**Table 3:** Representative microprobe analyses of apatite from the Niutishan mafic sill (in wt%).

**Sample point**	**Major zone**	**Height (m)**	**P**_**2**_**O**_**5**_	**SiO**_**2**_	**TiO**_**2**_	**Al**_**2**_**O**_**3**_	**FeO**	**MnO**	**MgO**	**CaO**	**Na**_**2**_**O**	**F**	**Cl**	**Total**	**-O=(F,Cl)**	**Total**	**OH**	**X**_**F**_
6-ap-1	*UZ*	-1.6	41.57	1.12	0.00	0.17	0.62	0.08	0.20	54.58	0.11	3.00	0.31	101.72	-1.33	100.23	1.00	0.95
10-ap-1	*MZ*	-3.6	40.47	0.81	0.00	0.07	0.54	0.02	0.10	55.79	0.02	3.40	0.19	101.41	-1.47	99.87	0.89	0.97
12-ap-1		-4.5	41.02	2.18	0.00	0.10	1.08	0.08	0.13	53.84	0.03	3.11	0.13	101.70	-1.34	100.26	1.02	0.98
18-ap-1		-7.5	40.28	0.88	0.02	0.27	0.76	0.06	0.11	54.61	0.11	3.32	0.17	100.60	-1.43	98.86	0.92	0.97
20-ap-1		-8.4	39.92	2.61	0.00	0.85	0.54	0.04	0.01	53.06	0.57	3.41	0.14	101.13	-1.47	98.82	0.90	0.98
22-ap-1		-10.8	40.37	4.25	0.00	1.05	0.21	0.07	0.03	52.38	0.24	3.51	0.20	102.30	-1.52	99.73	0.88	0.97
23-ap-1		-11.8	40.53	1.42	0.00	0.39	1.62	0.07	0.07	53.67	0.12	3.39	0.15	101.42	-1.46	101.00	0.88	0.98
24-ap-1		-13.3	41.16	2.55	0.00	0.96	0.74	0.04	0.08	53.33	0.51	3.25	0.15	102.75	-1.40	100.39	0.97	0.98
28-ap-1		-16.2	40.97	0.28	0.00	0.03	0.72	0.11	0.12	55.44	0.02	3.39	0.21	101.28	-1.49	100.25	0.90	0.97
30-ap-1	*LZc*	-17.2	41.82	0.25	0.01	0.06	0.59	0.12	0.06	55.83	0.00	3.13	0.17	102.04	-1.36	100.61	0.99	0.97

X_F_=molar F/(F + Cl)

**Table 4:** Microprobe analyses of primary Ti-magnetite and its exsolved ilmenite lamellae from the LZ of the Niutishan mafic sill (in wt%).

**Sample no.**	***NS-33 (LZb)***											
	33 -1		33-2		33-3		33-4		33-7		33-8	
	Mt-1	Ilm-1	Mt-2	Ilm-2	Mt-3	Ilm-3	Mt-4	Ilm-4	Mt-7	Ilm-7	Mt-8	Ilm-8
FeO	74.6	46.55	78.3	46.7	78.15	46.5	76.81	46.65	78.4	46.51	77.59	46.67
TiO_2_	16.19	50.77	12.53	51.03	11.99	50.93	14.92	50.9	13.04	51.24	13.73	51.25
V_2_O_3_	1.03	0.49	1.12	0.46	1.05	0.47	0.99	0.47	1.54	0.49	1.08	0.5
Cr_2_O_3_	0.01	0	0.01	0.01	0	0	0	0	0	0	0.04	0.02
NiO	0	0	0	0	0	0	0.03	0.02	0	0	0	0.01
CaO	0.12	0.08	0.13	0.09	0.2	0.12	0.08	0.11	0.1	0.13	0.07	0.1
MnO	0.63	1.7	0.48	1.7	0.55	1.72	0.57	1.78	0.54	1.74	0.54	1.64
Al_2_O_3_	0.98	0.02	1.1	0.03	1.76	0.03	0.9	0.02	0.55	0.02	1.42	0.02
Na_2_O	0.04	0	0.06	0	0.01	0	0.01	0	0	0.02	0.01	0
MgO	0.01	0.01	0	0.01	0	0.01	0.01	0.01	0.01	0.01	0	0.02
SiO_2_	0.1	0.03	0.09	0.01	0.1	0.03	0.06	0.03	0.08	0.04	0.09	0.04
Total	93.71	99.65	93.82	100.04	93.81	99.81	94.38	99.99	94.26	100.2	94.57	100.27
T(°C)^a^	652		593		586		636		569		593	
aTiO_2_^a^	0.23		0.22		0.22		0.23		0.19		0.20	
∆NNO^a^	-3.08		-3.10		-3.15		-3.01		-3.54		-3.4	

a Temperature and oxygen fugacity were calculated using the online MELTS software (http://melts.ofm-research.org). The calculations were performed using the model of Ghiorso and Evans (2008). NNO refers to the nickel-nickel oxide oxygen buffer, as calculated from O׳Neill and Pownceby (1993) using the pressure correction suggested by Frost (1991) with an assumed pressure of 200 MPa. The liquid activity of TiO_2_ is calculated by evaluating the equilibrium reaction: 2 FeTiO_3_ (component in ilmenite) = Fe_2_TiO_4_ (component in magnetite) + TiO_2_ (liquid component).

**Table 5:** Representative microprobe analyses of different types of ilmenite from the Niutishan mafic sill (in wt%).

**Sample no.**	**Major zone**	**TiO**_**2**_	**Cr**_**2**_**O**_**3**_	**FeO**	**MgO**	**MnO**	**NiO**	**Total**	**X**_**geik**_	**X**_**pyr**_	**X**_**hem**_	**X**_**ilm**_
**Euhedral - tabular**												
6-ilm-1	*UZ*	48.61	0.04	45.90	0.27	1.65	0.00	100.01	1.00	3.47	7.21	88.32
8-ilm-2	*UZ*	50.00	0.03	47.20	0.07	1.88	0.00	100.15	0.25	3.87	8.08	87.80
10-ilm-2	*MZ*	50.69	0.02	46.21	0.03	2.12	0.03	100.08	0.11	4.44	4.60	90.85
12-ilm-1	*MZ*	51.17	0.05	46.54	0.03	2.34	0.01	100.29	0.09	4.84	5.88	89.18
18-ilm-g2	*MZ*	50.31	0.15	47.11	0.05	2.29	0.00	100.71	0.19	4.69	8.23	86.89
22-ilm-g1	*MZ*	50.71	0.00	46.63	0.03	2.29	0.00	99.91	0.12	4.73	6.77	88.38
24-ilm-g1	*MZ*	52.04	0.02	46.58	0.01	2.26	0.00	101.05	0.03	4.68	4.11	91.18
32-ilm-g1	*LZb*	51.97	0.02	46.72	0.01	2.04	0.00	100.96	0.02	4.24	4.10	91.64
33-ilm-g1	*LZb*	50.59	0.04	47.25	0.02	1.71	0.01	100.24	0.09	3.53	5.80	90.59
35-ilm-g1	*LZa*	50.74	0.05	46.96	0.02	2.47	0.02	100.37	0.05	5.06	7.70	87.19
39-ilm-g3	*LZa*	50.77		47.02	0.07	1.96		99.99	0.07	4.58	7.34	88.02
37-ilm-g1	*LZa*	49.86	0.01	45.62	0.03	2.14	0.01	97.73	0.11	4.53	6.22	89.14
40-ilm-g1	*LZa*	49.84	0.01	46.94	0.07	2.04	0.00	99.06	0.18	4.45	5.78	89.58
**Acicular shape**												
12-ilm-l2	*MZ*	51.41	0.09	46.86	0.00	2.19	0.00	100.80	0.01	4.52	5.61	89.87
13-ilm-l2	*MZ*	51.18	0.00	46.65	0.03	2.22	0.02	100.47	0.11	4.59	5.56	89.73
20-ilm-l1	*MZ*	52.08	0.03	46.77	0.00	2.32	0.00	101.30	0.01	4.52	5.61	89.87
22-ilm-l1	*MZ*	50.25	0.00	46.45	0.07	2.17	0.01	100.42	0.24	4.51	5.86	89.39
32-ilm-l1	*LZb*	52.07	0.04	47.08	0.02	2.00	0.00	101.31	0.07	4.11	4.62	91.20
32-ilm-l2	*LZb*	51.53	0.00	46.51	0.00	1.94	0.01	100.05	0.00	4.06	4.27	91.68


**Sample no.**	**Magjor zone**	**TiO**_**2**_	**Cr**_**2**_**O**_**3**_	**FeO**	**MgO**	**MnO**	**NiO**	**Total**	**X**_**geik**_	**X**_**pyr**_	**X**_**hem**_	**X**_**ilm**_
**Comby-shape**												
8-ilm-f1	*UZ*	50.09		47.48	0.07	1.85		100.49	0.24	3.78	8.60	87.37
12-ilm-f4	*MZ*	49.22	0.05	46.49	0.06	2.27	0.00	99.66	0.23	4.70	8.57	86.50
15-ilm-f2	*MZ*	49.49	0.05	44.78	0.04	2.15	0.02	99.33	0.17	4.63	4.91	90.29
20-ilm-f1	*MZ*	50.46	0.06	46.91	0.02	2.29	0.00	100.40	0.08	4.72	7.49	87.71
29-ilm-f1	*MZ*	50.73	0.03	46.22	0.02	2.32	0.00	100.34	0.09	4.84	5.86	89.22
33-ilm-f1	*LZb*	49.74	0.05	47.27	0.19	1.75	0.00	99.94	0.68	3.58	8.29	87.44
36-ilm-f2	*LZa*	50.39		46.87	0.08	2.43		100.78	0.28	4.97	8.25	86.51
39-ilm-f1	*LZa*	50.33		46.83	0.05	2.26		99.72	0.17	4.64	7.81	87.38
**Rim on titanite**												
8-ilm-b1	*UZ*	50.13	0.06	47.04	0.07	1.84	0.00	100.58	0.27	3.83	4.74	91.16
8-ilm-b2	*UZ*	49.76		46.92	0.16	1.89		100.27	0.58	3.90	7.54	87.98
8-ilm-p1	*UZ*	48.85		44.85	0.17	1.78		100.33	0.63	3.84	5.78	89.74
8-ilm-p2	*UZ*	50.07		46.46	0.17	1.92		101.18	0.61	3.99	6.61	88.79
**Ilmenite lamellae**												
33-ilm-la2	*LZb*	50.10	0.03	47.54	0.12	1.71	0.00	100.34	0.55	3.80	6.51	89.14
36-ilm-la2	*LZa*	49.74		45.59	0.17	2.08		100.10	0.65	4.38	6.24	88.73
37-ilm4-la3	*LZa*	49.13	0.04	45.65	0.03	1.97	0.00	97.12	0.11	4.18	7.38	88.32
39-ilm-la2	*LZa*	50.01		45.45	0.14	2.16		99.65	0.51	4.56	4.60	90.32
**Ilmenite edge on tit-mag**												
36-ilm-e1	*LZa*	50.53	0.03	46.19	0.09	2.27	0.00	99.97	0.33	4.72	6.44	88.50

X_ilm_= 100[Fe^2+^/(Mn + Mg + Fe_T_)]; X_hem_=100[Fe^3+^/(Mn + Mg + Fe_T_)]; X_pyr_= 100[Mn/(Mn + Mg + Fe_T_)]; X_geik_=100[Mg/(Mn + Mg + Fe_T_)]. Fe_T_=Fe^3+^ + Fe^2+^.

Fe^3+^ and Fe^2+^ is calculated from stoichiometry.

**Table 6:** Representative microprobe analyses of titanite from the Niutishan mafic sill (in wt%).

Sample point	8-ttn-1	8-ttn-2	8-ttn-4-1	8-ttn-5	8-ttn-6	8-ttn-7	8-ttn-8	8-ttn-9	8-ttn-12	8-ttn-13	8-ttn-14	39-ttn-5		39-ttn-6
Type	Subhedral titanite (*NS-08, UZ*)											Tiny titanite (*NS-39, LZa*)		
Major elements (wt.%)														
SiO_2_	30.18	30.17	30.39	30.65	30.19	29.94	30.42	30.54	30.03	30.88	29.67		29.86	29.19
TiO_2_	34.91	34.82	35.36	35.22	34.45	33.47	32.56	34.00	32.24	33.39	35.77		34.85	36.49
Al_2_O_3_	2.35	2.24	1.72	1.98	2.16	2.37	2.43	2.60	2.42	2.49	1.71		2.70	1.83
CaO	27.74	27.77	27.79	27.63	27.01	26.78	26.23	27.05	26.15	26.82	28.10		27.05	27.67
MnO	0.03	0.07	0.02	0.03	0.04	0.02	0.05	0.03	0.06	0.05	0.06		0.05	0.07
Fe_2_O_3_	2.79	2.66	1.50	1.78	3.24	3.18	3.98	2.41	3.92	3.21	1.46		2.91	2.30
MgO	0.18	0.14	0.08	0.08	0.23	0.23	0.26	0.26	0.29	0.26	0.02		0.26	0.06
F	0.00	0.00	0.00	0.00	0.00	0.00	0.00	0.00	0.00	0.00	0.00		0.00	0.00
Cl	0.03	0.08	0.12	0.04	0.03	0.07	0.03	0.03	0.04	0.04	0.05		0.05	0.01
Total	98.22	97.95	96.97	97.41	97.35	96.05	95.95	96.91	95.15	97.14	96.85		97.73	97.62
**apfu.**														
Ca	0.99	1.00	1.00	0.98	0.97	0.98	0.96	0.97	0.97	0.96	1.02		0.97	1.00
Fe	0.07	0.05	0.04	0.07	0.08	0.08	0.10	0.06	0.08	0.08	0.04		0.07	0.06
Mg	0.01	0.00	0.00	0.01	0.01	0.01	0.01	0.01	0.01	0.01	0.00		0.01	0.00
Mn	0.00	0.00	0.00	0.00	0.00	0.00	0.00	0.00	0.00	0.00	0.00		0.00	0.00
Al	0.09	0.07	0.07	0.08	0.09	0.09	0.10	0.10	0.10	0.10	0.07		0.11	0.07
Ti	0.87	0.89	0.89	0.88	0.87	0.85	0.83	0.86	0.83	0.84	0.91		0.87	0.92
Si	1.01	1.02	1.02	1.01	1.01	1.02	1.03	1.03	1.04	1.04	1.00		1.00	0.98

**Table 7:** Representative titanite analyses (in ppm).

Sample point	8-ttn-1	σ	8-ttn-2	σ	8-ttn-4	σ	8-ttn-5	σ	8-ttn-6	σ	8-ttn-7	σ	8-ttn-8	σ
Type	Subhedral titanite (*NS-08, UZ*)													
Sr	25.90	0.99	20.06	1.31	21.34	0.85	18.80	0.84	19.77	0.82	18.30	0.81	19.52	1.26
Ta	4.66	0.20	2.60	0.22	3.02	0.14	4.71	0.25	3.65	0.18	2.53	0.14	1.76	0.15
Nb	55.93	2.19	42.45	3.00	66.40	2.69	64.09	2.91	39.36	1.67	48.26	2.23	39.71	2.78
Th	0.70	0.04	0.82	0.08	0.75	0.05	0.82	0.06	0.36	0.04	1.00	0.06	0.87	0.09
U	0.47	0.03	1.25	0.11	0.94	0.06	0.95	0.06	0.52	0.04	1.46	0.08	1.24	0.11
Zr	154.98	5.80	98.76	6.35	84.08	3.28	42.63	1.88	63.18	2.57	163.01	7.02	186.12	11.76
La	3.23	0.15	2.36	0.19	2.37	0.12	2.34	0.13	2.93	0.15	3.07	0.16	2.89	0.23
Ce	7.58	0.32	7.44	0.57	6.05	0.27	6.37	0.32	10.24	0.47	8.45	0.42	6.88	0.53
Pr	1.30	0.07	1.32	0.12	0.92	0.05	1.23	0.08	2.12	0.11	1.52	0.09	1.90	0.16
Nd	8.33	0.44	7.96	0.77	5.83	0.35	7.15	0.48	13.04	0.73	9.39	0.58	15.36	1.41
Sm	3.96	0.25	3.27	0.36	2.92	0.21	3.84	0.30	6.07	0.38	5.27	0.34	7.95	0.70
Eu	8.24	0.38	7.26	0.63	6.55	0.32	7.16	0.40	8.88	0.45	9.16	0.50	7.02	0.60
Gd	5.22	0.29	4.09	0.41	3.89	0.25	5.16	0.36	8.14	0.47	6.39	0.39	7.84	0.68
Tb	1.17	0.06	1.02	0.09	0.91	0.05	1.24	0.08	1.73	0.09	1.62	0.09	1.66	0.14
Dy	8.06	0.39	7.02	0.60	6.51	0.34	9.22	0.52	12.04	0.61	11.63	0.63	10.42	0.86
Ho	1.60	0.08	1.40	0.13	1.35	0.07	1.88	0.11	2.49	0.13	2.38	0.13	1.90	0.17
Er	3.84	0.20	3.42	0.32	3.54	0.20	4.65	0.29	5.96	0.33	6.07	0.35	4.52	0.41
Tm	0.54	0.03	0.50	0.06	0.46	0.03	0.66	0.05	0.74	0.05	0.83	0.06	0.51	0.06
Yb	3.31	0.20	2.97	0.32	3.00	0.20	4.26	0.30	4.08	0.27	5.16	0.33	3.72	0.38
Lu	0.28	0.03	0.25	0.04	0.35	0.03	0.41	0.04	0.40	0.04	0.42	0.03	0.38	0.05
T(°C)^a^	592		574		567		541		556		594		600	


Sample point	8-ttn-9	σ	8-ttn-12	σ	8-ttn-13	σ	8-ttn-14	σ
Type	Subhedral titanite (*NS-08, UZ*)							
Sr	23.78	1.32	17.91	0.91	18.90	0.85	24.10	1.17
Ta	3.48	0.24	3.77	0.23	4.09	0.22	2.21	0.14
Nb	51.87	3.10	57.75	3.12	57.03	2.68	42.02	2.17
Th	1.47	0.11	0.64	0.05	0.58	0.04	0.47	0.04
U	0.60	0.05	0.65	0.05	0.29	0.03	0.42	0.03
Zr	284.68	15.51	138.80	6.92	151.82	6.66	224.48	10.66
La	2.13	0.15	3.15	0.19	2.89	0.16	3.23	0.19
Ce	8.11	0.53	8.01	0.47	7.00	0.36	7.06	0.40
Pr	1.59	0.12	1.45	0.10	1.24	0.08	1.18	0.08
Nd	9.91	0.80	8.38	0.61	7.96	0.51	6.69	0.48
Sm	4.81	0.40	3.92	0.29	3.57	0.26	3.08	0.25
Eu	5.85	0.43	8.22	0.54	9.52	0.53	8.11	0.51
Gd	5.41	0.43	5.35	0.37	4.37	0.29	4.83	0.34
Tb	1.10	0.08	1.20	0.08	0.96	0.06	0.95	0.06
Dy	8.13	0.59	7.80	0.50	6.46	0.38	6.53	0.42
Ho	1.70	0.13	1.61	0.11	1.42	0.09	1.29	0.09
Er	4.57	0.36	3.87	0.27	3.45	0.22	3.53	0.24
Tm	0.58	0.05	0.47	0.04	0.46	0.04	0.43	0.04
Yb	3.66	0.32	2.80	0.22	2.77	0.20	3.12	0.24
Lu	0.46	0.05	0.31	0.03	0.29	0.03	0.30	0.03
T(°C)	618		588		591		608	

a Zr-in-titanite temperatures were calculated for a quartz- and titanite-bearing, rutile-absent sample (NS-08) using the Hayden et al. [7] calibration

## 2 Experimental design, materials, and methods

### 2.1 Sampling

A total of 38 samples were collected systematically with a constant spacing of ~0.5 m from the top to the bottom of the Niutishan mafic sill. The true stratigraphic position and the distance between samples were corrected based on the average local dip (17° SE). The real outcrop thickness of this sill is ~22 m, and the top surface was set to ‘0 m’. The erosion surface of sample was carefully removed. All analytical procedures were accomplished at the Institute of Geology and Geophysics, Chinese Academy of Sciences (IGGCAS).

### 2.2 Whole-rock analysis

Sample powders, ~0.5 g for each, were fused into a glass bead with ~5.0 g of lithium tetraborate. Major elements were measured using a PANalytical AXIOS Minerals instrument, and ferrous iron was measured by titration [2] (Table S1). The uncertainties for major elements were<1.0 wt%. The precision was better than 0.2 wt% in the analytical range. The loss-on-ignition (LOI) was determined by the weight loss for a powdered sample after 2 h heating at 1000 °C and 30 min cooling to ambient temperature. Two reference materials GSR1 and GSR3 were measured to evaluate the chemical preparation procedure and the condition of the instrument. GSR1 and GSR3 data are listed in Table S1, and are consistent with the reference values.

Whole-rock trace element analyses were determined using an ELEMENT instrument after HNO_3_ + HF digestion of approximately 40 mg of sample powder in a Teflon vessel, with accuracy and reproducibility monitored using Chinese national standard samples GSR1 and GSR3. GSR1 and GSR3 data are listed in Table S1 and are consistent with the reference values. Both precision and accuracy are better than 5% for most of the trace elements (Table S1).

### 2.3 Electron microprobe mapping and analysis

Thin and polished sections of the gabbroic rocks that were coated with carbon were made for the analysis of major elements and backscattered electron images. Major element compositions of plagioclase, clinopyroxene, apatite and ilmenite were obtained by a JEOL JXA 8100 instrument (an electron microprobe analysis). Quantitative analyses were performed using wavelength-dispersive spectrometers (WDS) with an acceleration voltage of 15 kV, a beam current of 20 nA, and a defocused beam size of 5 μm. Major element compositions of titanite, titano-magnetite and ilmenite lamellae were measured by CAMECA SX Five FE instrument (an electron probe micro-analyser). Quantitative analyses were performed using wavelength-dispersive spectrometers (WDS) with an acceleration voltage of 15 kV, a beam current of 30 nA, and a focused beam size (0 μm). The peak counting time was 10–20 s for all elements, and the background counting time was 10 s on the high- and low-energy background positions. All data were corrected online using a modified ZAF (atomic number, absorption, fluorescence) correction procedure. The detection limits were in the range of 0.008–0.02 wt% (1σ). The accuracy was generally less than 1 wt% for oxide contents greater than 5 wt% (Table 1, Table 2, Table 3, Table 4, Table 5, Table 6).

### 2.4 Fe-Ti two-oxide geothermometer and oxygen barometer

There are a few preserved coexisting titano-magnetite and ilmenite grains in the thin sections, due to the exsolved mature of ilmenite in oxide pairs. The titano-magnetite and nearly coeval thick ilmenite lamellae led to our adoption of a revised Fe-Ti two-oxide geothermometer and oxygen barometer. Temperature and oxygen fugacity were calculated using the online MELTS software (http://melts.ofm-research.org). The calculations were performed using the model of Ghiorso and Evans [3]. The results include the Fe-Ti exchange temperature, *fO*_2_ (relative to NNO) and aTiO_2_ (liquid, relative to rutile saturation). NNO refers to the nickel-nickel oxide oxygen buffer, as calculated from O׳Neill and Pownceby [4] using the pressure correction suggested by Frost [5] with an assumed pressure of 200 MPa. The liquid activity of TiO_2_ is calculated by evaluating the equilibrium reaction: 2 FeTiO_3_ (component in ilmenite) = Fe_2_TiO_4_ (component in magnetite) + TiO_2_ (liquid component). The results are shown in Table 4.

### 2.5 Titanite LA-ICP-MS trace element analysis and Zr-in-titanite thermometry

Titanite trace elements were analysed by using an LA-ICP-MS system composed of an Agilent 7500a ICP-MS coupled with a Resonetic RESOLution M–50 ArF–Excimer laser source (λ = 193 nm). The pulse width was 15 ns. The laser energy was 98 mJ, with a repetition rate of 8 Hz, spot sizes of 44 μm and 60 μm in diameter and a total of 45 s ablation time. Helium was used as the carrier gas to enhance the transport efficiency of the ablated material. Trace concentrations were calibrated by using 29Si as an internal standard and NIST SRM 610 as the reference standard. The element concentrations were measured using the GLITTER 4.0 software [6] for elemental fraction correction. The data after calibration (1σ) are shown in Table 7.

A powerful tool to calculate the crystallisation temperature is the Zr-in-titanite thermometer. Zr-in-titanite temperatures were calculated for a quartz- and titanite-bearing, rutile-absent sample (NS-08) using the Hayden et al. [7] calibration:

$$T\left({}^{\circ}C\right)=\frac{\left[7708\;+\;960P\left(\mathrm{GPa}\right)\right]}{\left[10.52\;-\;\log\left(a_{\mathrm{Ti}O_{2}}\right)-\;\log\left(a_{\mathrm{Si}O_{2}}\right)\;-\;\log\left(\mathrm{ppm Zr},\mathrm{titanite}\right)\right]}\;-\;273$$

where $a_{\mathrm{Si}O_{2}}$ = 1 and $a_{\mathrm{Ti}O_{2}}=0.22\pm 0.01$ (2σ, n = 6); pressure is based on the total Neoproterozoic sedimentary strata thickness overlaying the Niutishan sill, corresponding to the lithostatic pressure at a depth of 2.2 km. Reported temperatures in Table 7 are the weighted average of multiple spots from a single sample.

## Footnotes

Transparency document Transparency document associated with this article can be found in the online version at https://doi.org/10.1016/j.dib.2018.10.013.
