# Supplementary material for: Whole-rock and mineral chemical data from a profile of the ~900 Ma Niutishan Fe-Ti-rich sill in XuZhou, North China
Source: Data Brief. 2018 Oct 9;21:727–35. doi: 10.1016/j.dib.2018.10.013 (PMC6214829; doi:10.1016/j.dib.2018.10.013)
Supplement: Supplementary file 1 — Transparency document [file mmc1.docx]

Conflict of interest

All authors confirms as No conflict of Interest.
