# Supplementary material for: Whole-rock and mineral chemical data from a profile of the ~900 Ma Niutishan Fe-Ti-rich sill in XuZhou, North China
Source: Data Brief. 2018 Oct 9;21:727–35. doi: 10.1016/j.dib.2018.10.013 (PMC6214829; doi:10.1016/j.dib.2018.10.013)
Supplement: Supplementary file 2 — Supplementary material [file mmc2.docx]

|  | ***Reference materials*** | | | |  |  | ***CM*** |  |  | ***UZ*** |
| --- | --- | --- | --- | --- | --- | --- | --- | --- | --- | --- |
| Sample: | **GSR1^a^** | **SD^b^** | **GSR3^a^** | **SD^b^** | **GSR1^c^** | **GSR3^c^** | **NS-01** | **NS-02** | **NS-03** | **NS-04** |
| Depth(m) |  |  |  |  |  |  | -0.02 | -0.25 | -0.54 | -0.81 |
| SiO_2_ | *72.83* | *0.15* | *44.64* | *0.16* | *73.21* | *44.63* | *48.95* | *48.83* | *48.68* | *48.81* |
| TiO_2_ | *0.29* | *0.01* | *2.37* | *0.06* | *0.29* | *2.37* | *2.90* | *2.90* | *2.87* | *2.89* |
| Al_2_O_3_ | *13.40* | *0.11* | *13.83* | *0.20* | *13.57* | *13.69* | *12.71* | *12.79* | *12.65* | *12.66* |
| TFe_2_O_3_ | *2.14* | *0.08* | *13.4* | *0.29* | *2.11* | *13.32* | *15.62* | *15.75* | *15.84* | *15.85* |
| MnO | *0.060* | *0.003* | *0.169* | *0.009* | *0.060* | *0.170* | *0.24* | *0.23* | *0.24* | *0.23* |
| MgO | *0.42* | *0.05* | *7.77* | *0.26* | *0.42* | *7.66* | *5.04* | *5.00* | *5.10* | *5.01* |
| CaO | *1.55* | *0.07* | *8.81* | *0.14* | *1.56* | *8.77* | *8.91* | *8.71* | *8.79* | *8.79* |
| Na_2_O | *3.13* | *0.09* | *3.38* | *0.07* | *3.14* | *3.37* | *3.40* | *3.16* | *3.33* | *3.15* |
| K_2_O | *5.01* | *0.10* | *2.32* | *0.08* | *5.02* | *2.29* | *0.51* | *0.74* | *0.61* | *0.70* |
| P_2_O_5_ | *0.093* | *0.003* | *0.946* | *0.019* | *0.092* | *0.933* | *0.30* | *0.30* | *0.29* | *0.30* |
| LOI | *0.70* |  | *2.24* |  | *0.60* | *2.30* | *2.02* | *1.98* | *1.92* | *1.84* |
| Total | *99.62* |  | *99.86* |  | *100.07* | *99.49* | *100.60* | *100.39* | *100.32* | *100.23* |
| FeO | *1.02* | *0.06* | *7.60* | *0.16* |  | *7.75* | *11.16* | *11.74* | *12.27* | *12.09* |
| Mg# | *27.89* |  | *53.30* |  |  | *53.09* | *38.80* | *38.41* | *38.74* | *38.30* |
| Th | 54 | 4 | 6 | 1.2 | 55.24 | 6.46 | 2.95 |  | 3.15 | 2.80 |
| U | 18.8 | 2.2 | 1.4 | 0.4 | 18.20 | 1.48 | 0.61 |  | 0.66 | 0.64 |
| Nb | 40 | 4 | 68 | 12 | 39.77 | 68.91 | 15.54 |  | 16.44 | 15.22 |
| Ta | 7.2 | 0.7 | 4.3 | 0.6 | 6.95 | 4.68 | 1.07 |  | 1.14 | 1.06 |
| Sr | 106 | 9 | 1100 | 100 | 114.80 | 1121.56 | 245.07 |  | 262.11 | 251.48 |
| Zr | 167 | 14 | 277 | 30 | 178.91 | 275.34 | 183.75 |  | 193.81 | 177.04 |
| Hf | 6.3 | 0.8 | 6.5 | 0.8 | 6.01 | 6.86 | 5.19 |  | 5.38 | 5.18 |
| Y | 62 | 7 | 22 | 5 | 63.17 | 21.64 | 35.94 |  | 38.57 | 35.94 |
| V | 24 | 3 | 167 | 17 | 27.26 | 164.94 | 406.44 |  | 433.38 | 392.72 |
| Cr | 3.6 | 1.1 | 134 | 16 | 4.50 | 132.03 | 61.95 |  | 69.92 | 63.54 |
| Co | 3.4 | 1.0 | 46.5 | 5.2 | 3.70 | 45.49 | 43.26 |  | 47.34 | 43.95 |
| Ni | 2.3 | 1.2 | 140 | 11 | 0.34 | 135.21 | 38.27 |  | 38.39 | 34.87 |
| La | 54 | 5 | 56 | 7 | 53.90 | 57.81 | 18.92 |  | 19.84 | 18.72 |
| Ce | 108 | 11 | 105 | 12 | 108.03 | 110.28 | 41.96 |  | 44.17 | 41.57 |
| Pr | 12.7 | 0.8 | 13.2 | 1.6 | 13.07 | 13.44 | 6.09 |  | 6.40 | 6.00 |
| Nd | 47 | 5 | 54 | 5 | 48.25 | 55.28 | 26.31 |  | 27.86 | 26.56 |
| Sm | 9.7 | 1.2 | 10.2 | 0.7 | 10.19 | 10.69 | 6.82 |  | 7.29 | 7.00 |
| Eu | 0.85 | 0.10 | 3.2 | 0.3 | 0.85 | 3.22 | 2.23 |  | 2.36 | 2.27 |
| Gd | 9.3 | 0.8 | 8.5 | 0.7 | 8.89 | 9.15 | 7.25 |  | 7.81 | 7.25 |
| Tb | 1.65 | 0.13 | 1.2 | 0.2 | 1.69 | 1.25 | 1.20 |  | 1.29 | 1.20 |
| Dy | 10.2 | 0.4 | 5.6 | 0.3 | 11.00 | 5.72 | 7.42 |  | 7.93 | 7.63 |
| Ho | 2.05 | 0.22 | 0.88 | 0.05 | 2.20 | 0.99 | 1.50 |  | 1.60 | 1.52 |
| Er | 6.5 | 0.4 | 2 | 0.3 | 6.67 | 2.08 | 4.09 |  | 4.35 | 4.03 |
| Tm | 1.06 | 0.11 | 0.28 | 0.04 | 1.09 | 0.25 | 0.59 |  | 0.62 | 0.57 |
| Yb | 7.4 | 0.7 | 1.5 | 0.5 | 7.60 | 1.38 | 3.68 |  | 3.95 | 3.59 |
| Lu | 1.15 | 0.12 | 0.19 | 0.07 | 1.21 | 0.19 | 0.55 | 0.58 | 0.59 | 0.59 |

Table 1: Major (wt.%) and trace (ppm) elements data of the Niutishan mafic sill

Table 1: Continued

|  | ***UZ*** |  |  | ***MZ*** |  |  |  |  |  |  |
| --- | --- | --- | --- | --- | --- | --- | --- | --- | --- | --- |
| Sample: | **NS-06** | **NS-08** | **NS-09** | **NS-10** | **NS-11** | **NS-12** | **NS-12^d^** | **NS-13** | **NS-14** | **NS-15** |
| Depth(m) | -1.63 | -2.60 | -3.08 | -3.57 | -4.06 | -4.54 |  | -5.02 | -5.51 | -6 |
| SiO_2_ | *48.67* | *48.44* | *48.51* | *50.33* | *53.33* | *52.81* | *52.29* | *53.18* | *52.24* | *53.81* |
| TiO_2_ | *2.86* | *3.02* | *3.28* | *3.32* | *2.35* | *2.44* | *2.416* | *2.33* | *2.48* | *2.11* |
| Al_2_O_3_ | *12.70* | *12.27* | *12.14* | *12.07* | *12.12* | *11.98* | *11.93* | *12.36* | *11.79* | *12.17* |
| TFe_2_O_3_ | *15.60* | *16.54* | *16.88* | *17.05* | *15.78* | *16.27* | *16.21* | *17.46* | *16.64* | *15.84* |
| MnO | *0.22* | *0.24* | *0.27* | *0.28* | *0.22* | *0.24* | *0.23* | *0.22* | *0.25* | *0.23* |
| MgO | *4.92* | *4.82* | *4.71* | *3.13* | *2.25* | *2.45* | *2.43* | *2.12* | *2.47* | *2.12* |
| CaO | *8.77* | *8.73* | *7.90* | *6.94* | *5.94* | *6.69* | *6.61* | *3.65* | *6.24* | *5.91* |
| Na_2_O | *3.03* | *2.74* | *3.09* | *3.17* | *3.68* | *3.57* | *3.5268* | *4.34* | *3.40* | *3.60* |
| K_2_O | *0.78* | *0.87* | *0.94* | *1.11* | *1.04* | *0.79* | *0.7883* | *0.86* | *0.96* | *0.93* |
| P_2_O_5_ | *0.30* | *0.32* | *0.35* | *0.48* | *0.60* | *0.59* | *0.5882* | *0.65* | *0.59* | *0.66* |
| LOI | *2.00* | *2.18* | *2.32* | *2.12* | *2.80* | *2.56* | *2.52* | *3.02* | *3.26* | *2.24* |
| Total | *99.84* | *100.16* | *100.38* | *99.99* | *100.10* | *100.39* | *99.5393* | *100.18* | *100.32* | *99.62* |
| FeO | *11.74* | *12.63* | *13.63* | *13.68* | *10.29* | *11.32* | *11.35* | *11.37* | *12.00* | *11.35* |
| Mg# | *38.25* | *36.40* | *35.39* | *26.49* | *21.90* | *22.84* | *22.76* | *19.28* | *22.58* | *20.82* |
| Th | 3.23 | 2.94 | 3.32 | 4.83 | 6.49 | 6.03 | 6.31 | 6.43 | 6.08 | 7.08 |
| U | 0.66 | 0.63 | 0.71 | 1.02 | 1.43 | 1.30 | 1.39 | 1.38 | 1.33 | 1.49 |
| Nb | 15.90 | 17.12 | 18.32 | 24.13 | 30.40 | 30.02 | 29.77 | 29.68 | 27.78 | 31.79 |
| Ta | 1.17 | 1.12 | 1.26 | 1.74 | 2.10 | 2.05 | 2.16 | 2.10 | 1.99 | 2.26 |
| Sr | 219.14 | 231.18 | 219.72 | 213.11 | 155.48 | 122.04 | 121.59 | 103.64 | 165.51 | 169.23 |
| Zr | 185.57 | 191.12 | 209.32 | 279.62 | 373.46 | 348.63 | 350.70 | 373.37 | 352.57 | 389.40 |
| Hf | 5.60 | 5.24 | 5.92 | 8.16 | 10.73 | 9.87 | 10.61 | 10.85 | 10.39 | 11.74 |
| Y | 37.13 | 38.66 | 42.40 | 52.97 | 68.18 | 65.18 | 65.11 | 66.19 | 64.04 | 73.34 |
| V | 400.29 | 418.46 | 482.74 | 248.41 | 89.87 | 85.13 | 84.86 | 79.89 | 101.99 | 58.60 |
| Cr | 64.20 | 70.65 | 48.61 | 33.78 | 76.49 | 70.94 | 66.99 | 64.28 | 48.57 | 51.79 |
| Co | 43.40 | 43.90 | 44.57 | 32.82 | 26.80 | 23.92 | 23.90 | 32.67 | 26.79 | 23.58 |
| Ni | 34.09 | 30.08 | 30.29 | 2.13 | 1.71 | 1.54 | 1.64 | 0.72 | 0.62 | 1.41 |
| La | 19.54 | 19.74 | 22.15 | 29.85 | 39.04 | 38.36 | 37.76 | 38.57 | 35.92 | 43.71 |
| Ce | 43.58 | 44.40 | 49.37 | 66.93 | 86.26 | 83.98 | 83.22 | 84.97 | 79.83 | 97.39 |
| Pr | 6.25 | 6.39 | 7.19 | 9.61 | 12.58 | 12.10 | 12.03 | 12.31 | 11.67 | 14.09 |
| Nd | 27.34 | 27.61 | 31.61 | 42.15 | 54.71 | 52.43 | 52.36 | 53.19 | 50.90 | 60.11 |
| Sm | 7.07 | 7.11 | 8.10 | 10.43 | 13.68 | 13.09 | 13.04 | 13.25 | 12.74 | 14.88 |
| Eu | 2.30 | 2.33 | 2.64 | 3.20 | 4.23 | 3.89 | 3.94 | 3.52 | 3.80 | 4.46 |
| Gd | 7.70 | 7.66 | 8.88 | 11.24 | 14.58 | 14.04 | 14.10 | 14.18 | 13.66 | 15.70 |
| Tb | 1.29 | 1.28 | 1.47 | 1.94 | 2.39 | 2.35 | 2.43 | 2.40 | 2.35 | 2.75 |
| Dy | 7.94 | 8.03 | 8.96 | 11.50 | 14.64 | 13.83 | 14.15 | 14.18 | 13.89 | 15.99 |
| Ho | 1.59 | 1.62 | 1.84 | 2.38 | 3.03 | 2.74 | 2.85 | 2.96 | 2.92 | 3.21 |
| Er | 4.33 | 4.39 | 4.85 | 6.12 | 7.94 | 7.47 | 7.78 | 7.73 | 7.56 | 8.71 |
| Tm | 0.62 | 0.63 | 0.69 | 0.90 | 1.12 | 1.06 | 1.11 | 1.10 | 1.07 | 1.23 |
| Yb | 3.85 | 3.88 | 4.23 | 5.46 | 6.83 | 6.51 | 6.71 | 6.77 | 6.44 | 7.52 |
| Lu | 0.59 | 0.59 | 0.64 | 0.82 | 1.02 | 0.98 | 1.03 | 1.02 | 0.99 | 1.15 |

Table 1: Continued

|  | ***MZ*** |  |  |  |  |  |  |  |  |  |
| --- | --- | --- | --- | --- | --- | --- | --- | --- | --- | --- |
| Sample: | **NS-16** | **NS-17** | **NS-18** | **NS-19** | **NS-20** | **NS-21** | **NS-22** | **NS-22^d^** | **NS-23** | **NS-24** |
| Depth(m) | -6.48 | -6.96 | -7.45 | -7.93 | -8.42 | -9.39 | -10.84 |  | -11.81 | -13.27 |
| SiO_2_ | *53.71* | *53.44* | *53.39* | *54.49* | *53.84* | *53.38* | *54.33* | *54.66* | *52.79* | *54.40* |
| TiO_2_ | *2.30* | *2.33* | *2.36* | *2.20* | *2.25* | *2.29* | *2.28* | *2.30* | *2.73* | *2.26* |
| Al_2_O_3_ | *12.32* | *12.07* | *12.14* | *12.26* | *12.25* | *12.20* | *12.16* | *12.17* | *12.09* | *11.86* |
| TFe_2_O_3_ | *16.20* | *15.81* | *16.55* | *15.60* | *15.94* | *16.53* | *15.29* | *15.34* | *16.70* | *16.47* |
| MnO | *0.22* | *0.24* | *0.25* | *0.25* | *0.25* | *0.25* | *0.25* | *0.25* | *0.26* | *0.24* |
| MgO | *2.17* | *2.27* | *2.20* | *2.15* | *2.30* | *2.40* | *2.12* | *2.13* | *2.73* | *2.27* |
| CaO | *5.38* | *6.10* | *6.23* | *6.45* | *6.41* | *5.91* | *6.35* | *6.41* | *6.65* | *5.22* |
| Na_2_O | *3.62* | *3.54* | *3.49* | *3.60* | *3.50* | *3.48* | *3.46* | *3.47* | *3.24* | *3.96* |
| K_2_O | *0.90* | *0.88* | *0.96* | *1.01* | *1.03* | *1.06* | *1.10* | *1.11* | *1.18* | *0.89* |
| P_2_O_5_ | *0.66* | *0.62* | *0.61* | *0.66* | *0.62* | *0.60* | *0.63* | *0.63* | *0.53* | *0.64* |
| LOI | *2.62* | *2.42* | *2.00* | *1.90* | *2.06* | *2.12* | *1.88* | *1.86* | *1.76* | *2.36* |
| Total | *100.09* | *99.71* | *100.18* | *100.56* | *100.45* | *100.22* | *99.86* | *100.34* | *100.66* | *100.56* |
| FeO | *10.70* | *11.04* | *12.28* | *11.61* | *11.01* | *11.90* | *10.43* | *10.47* | *12.68* | *11.28* |
| Mg# | *20.85* | *22.01* | *20.71* | *21.31* | *22.10* | *22.20* | *21.42* | *21.44* | *24.31* | *21.32* |
| Th | 6.76 | 6.88 | 6.83 | 7.56 |  | 6.09 | 6.34 |  | 6.56 | 6.33 |
| U | 1.34 | 1.48 | 1.39 | 1.56 |  | 1.21 | 1.36 |  | 1.36 | 1.36 |
| Nb | 31.78 | 32.42 | 34.62 | 35.23 |  | 27.14 | 31.73 |  | 31.74 | 32.24 |
| Ta | 2.12 | 2.23 | 2.26 | 2.38 |  | 1.82 | 2.14 |  | 2.18 | 2.16 |
| Sr | 170.59 | 176.76 | 193.00 | 195.96 |  | 163.28 | 220.77 |  | 342.96 | 153.79 |
| Zr | 388.53 | 395.50 | 404.39 | 434.64 |  | 351.27 | 371.52 |  | 388.61 | 377.16 |
| Hf | 10.92 | 11.47 | 11.18 | 12.42 |  | 9.81 | 10.70 |  | 11.08 | 10.69 |
| Y | 69.90 | 71.83 | 76.35 | 80.27 |  | 65.91 | 69.31 |  | 69.44 | 70.49 |
| V | 78.80 | 81.60 | 96.76 | 69.83 |  | 86.37 | 70.41 |  | 148.72 | 64.60 |
| Cr | 65.65 | 74.06 | 55.48 | 52.24 |  | 51.47 | 53.93 |  | 49.17 | 63.30 |
| Co | 27.17 | 25.01 | 26.24 | 26.54 |  | 27.08 | 20.78 |  | 33.57 | 30.09 |
| Ni | 0.42 | 0.56 | 0.53 | 0.24 |  | 5.84 | 0.77 |  | 1.79 | 0.38 |
| La | 42.10 | 42.41 | 43.02 | 47.93 |  | 38.67 | 39.76 |  | 37.89 | 38.41 |
| Ce | 94.31 | 96.44 | 98.27 | 107.32 |  | 83.55 | 90.28 |  | 87.57 | 86.99 |
| Pr | 13.31 | 13.59 | 14.09 | 15.15 |  | 12.12 | 12.74 |  | 12.44 | 12.42 |
| Nd | 59.05 | 58.49 | 65.97 | 66.47 |  | 52.54 | 55.61 |  | 54.72 | 55.40 |
| Sm | 14.49 | 14.69 | 15.74 | 16.39 |  | 13.08 | 14.14 |  | 14.07 | 13.85 |
| Eu | 4.24 | 4.26 | 4.50 | 5.02 |  | 3.81 | 4.23 |  | 4.19 | 3.77 |
| Gd | 14.98 | 15.01 | 15.90 | 16.92 |  | 13.75 | 14.55 |  | 14.23 | 14.53 |
| Tb | 2.50 | 2.61 | 2.71 | 2.86 |  | 2.31 | 2.46 |  | 2.44 | 2.45 |
| Dy | 14.55 | 15.25 | 15.85 | 16.90 |  | 13.53 | 14.49 |  | 15.11 | 14.55 |
| Ho | 3.04 | 3.22 | 3.19 | 3.42 |  | 2.73 | 2.91 |  | 3.21 | 3.02 |
| Er | 7.91 | 8.41 | 8.53 | 9.23 |  | 7.49 | 7.87 |  | 8.30 | 7.87 |
| Tm | 1.09 | 1.17 | 1.18 | 1.27 |  | 1.03 | 1.08 |  | 1.18 | 1.09 |
| Yb | 6.79 | 7.33 | 7.49 | 8.01 |  | 6.51 | 6.86 |  | 7.32 | 6.92 |
| Lu | 1.02 | 1.12 | 1.15 | 1.26 |  | 0.98 | 1.04 |  | 1.14 | 1.04 |

Table 1: Continued

|  | ***MZ*** |  |  |  |  |  | ***LZc*** | ***LZb*** |  |  |
| --- | --- | --- | --- | --- | --- | --- | --- | --- | --- | --- |
| Sample: | **NS-34** | **NS-35** | **NS-36** | **NS-37** | **NS-29** | **NS-29^d^** | **NS-31** | **NS-32** | **NS-33** | **NS-33^d^** |
| Depth(m) | -18.66 | -19.43 | -19.91 | -20.39 | -16.66 |  | -17.21 | -17.69 | -18.18 |  |
| SiO_2_ | *46.33* | *50.08* | *49.48* | *48.77* | *56.11* |  | *52.95* | *48.41* | *42.50* | *42.52* |
| TiO_2_ | *3.30* | *2.58* | *2.35* | *2.70* | *1.99* |  | *2.26* | *3.89* | *4.60* | *4.59* |
| Al_2_O_3_ | *11.87* | *13.30* | *12.98* | *13.12* | *12.11* |  | *12.22* | *11.60* | *11.25* | *11.26* |
| TFe_2_O_3_ | *19.10* | *13.56* | *14.61* | *14.18* | *15.04* |  | *17.06* | *18.33* | *24.53* | *24.51* |
| MnO | *0.25* | *0.21* | *0.21* | *0.21* | *0.25* |  | *0.28* | *0.27* | *0.26* | *0.26* |
| MgO | *4.93* | *5.09* | *5.10* | *5.17* | *1.87* |  | *1.97* | *3.98* | *4.51* | *4.52* |
| CaO | *8.31* | *9.47* | *9.50* | *10.05* | *5.59* |  | *6.03* | *7.86* | *7.37* | *7.37* |
| Na_2_O | *2.44* | *2.97* | *2.65* | *2.87* | *3.79* |  | *3.51* | *3.14* | *2.24* | *2.24* |
| K_2_O | *0.97* | *1.06* | *1.01* | *0.84* | *0.96* |  | *0.95* | *0.64* | *0.83* | *0.88* |
| P_2_O_5_ | *0.24* | *0.27* | *0.27* | *0.25* | *0.70* |  | *0.85* | *0.38* | *0.24* | *0.24* |
| LOI | *1.94* | *1.74* | *1.78* | *1.90* | *1.98* |  | *2.08* | *1.98* | *1.76* | *1.76* |
| Total | *99.68* | *100.33* | *99.95* | *100.06* | *100.39* |  | *100.15* | *100.48* | *100.09* | *100.10* |
| FeO | *12.47* | *10.05* | *10.60* | *10.22* | *10.47* |  | *11.72* | *13.33* | *14.84* | *14.83* |
| Mg# | *33.67* | *42.44* | *40.68* | *41.74* | *19.64* |  | *18.50* | *29.91* | *26.57* | *26.62* |
| Th | 3.18 | 2.54 | 2.48 | 2.26 | 7.86 | 7.62 | 6.47 | 3.94 | 2.86 |  |
| U | 0.61 | 0.55 | 0.52 | 0.49 | 1.65 | 1.60 | 1.31 | 0.87 | 0.58 |  |
| Nb | 14.84 | 14.93 | 13.33 | 13.41 | 34.10 | 33.61 | 31.53 | 22.59 | 15.96 |  |
| Ta | 1.02 | 0.99 | 0.89 | 0.87 | 2.33 | 2.34 | 2.13 | 1.62 | 1.15 |  |
| Sr | 237.77 | 185.99 | 277.32 | 181.91 | 193.48 | 186.43 | 198.43 | 183.66 | 234.32 |  |
| Zr | 181.69 | 159.23 | 154.61 | 146.62 | 452.96 | 439.69 | 355.26 | 255.94 | 180.55 |  |
| Hf | 5.25 | 4.52 | 4.39 | 4.14 | 13.01 | 12.68 | 10.10 | 7.40 | 5.28 |  |
| Y | 36.87 | 33.67 | 34.11 | 31.83 | 80.76 | 78.90 | 70.63 | 48.96 | 35.24 |  |
| V | 808.42 | 374.17 | 396.69 | 440.35 | 33.49 | 30.11 | 39.07 | 397.74 | 1231.86 |  |
| Cr | 50.98 | 110.99 | 104.98 | 122.94 | 60.14 | 61.13 | 74.81 | 26.26 | 27.89 |  |
| Co | 62.71 | 37.46 | 41.89 | 39.56 | 24.81 | 23.36 | 26.37 | 50.63 | 96.40 |  |
| Ni | 33.49 | 34.22 | 38.07 | 39.69 | 0.36 | 0.83 | 2.04 | 1.98 | 76.67 |  |
| La | 18.10 | 16.72 | 17.12 | 15.48 | 44.51 | 43.95 | 39.47 | 24.62 | 16.89 |  |
| Ce | 41.58 | 36.95 | 37.74 | 34.09 | 100.59 | 100.91 | 89.50 | 57.08 | 38.11 |  |
| Pr | 6.05 | 5.30 | 5.44 | 4.93 | 14.64 | 14.57 | 13.08 | 8.19 | 5.60 |  |
| Nd | 26.54 | 23.06 | 23.19 | 21.63 | 64.09 | 63.81 | 58.25 | 35.85 | 24.95 |  |
| Sm | 7.46 | 6.07 | 6.18 | 5.80 | 16.33 | 16.18 | 15.11 | 9.63 | 6.72 |  |
| Eu | 2.30 | 2.06 | 2.05 | 1.92 | 4.62 | 4.50 | 4.08 | 2.97 | 2.19 |  |
| Gd | 7.61 | 6.49 | 6.68 | 6.13 | 16.58 | 16.47 | 15.25 | 9.87 | 6.96 |  |
| Tb | 1.28 | 1.13 | 1.16 | 1.06 | 2.85 | 2.86 | 2.63 | 1.67 | 1.17 |  |
| Dy | 8.13 | 6.58 | 6.84 | 6.42 | 17.22 | 17.07 | 15.52 | 10.60 | 7.41 |  |
| Ho | 1.69 | 1.37 | 1.43 | 1.34 | 3.44 | 3.43 | 3.22 | 2.24 | 1.51 |  |
| Er | 4.60 | 3.65 | 3.74 | 3.46 | 9.47 | 9.47 | 8.25 | 5.81 | 4.11 |  |
| Tm | 0.66 | 0.52 | 0.53 | 0.50 | 1.32 | 1.30 | 1.15 | 0.82 | 0.58 |  |
| Yb | 4.17 | 3.30 | 3.35 | 3.17 | 8.28 | 8.11 | 7.18 | 5.15 | 3.78 |  |
| Lu | 0.64 | 0.49 | 0.50 | 0.47 | 1.29 | 1.23 | 1.11 | 0.78 | 0.58 |  |

Table 1: Continued

|  | ***LZb*** | ***LZa*** |  |  |  |  |  |
| --- | --- | --- | --- | --- | --- | --- | --- |
| Sample: | **NS-34** | **NS-35** | **NS-36** | **NS-37** | **NS-38** | **NS-39** | **NS-40** |
| Depth(m) | -18.66 | -19.43 | -19.91 | -20.39 | -20.87 | -21.35 | -21.83 |
| SiO_2_ | *46.33* | *50.08* | *49.48* | *48.77* | *48.17* | *50.12* | *49.52* |
| TiO_2_ | *3.30* | *2.58* | *2.35* | *2.70* | *2.94* | *2.44* | *2.36* |
| Al_2_O_3_ | *11.87* | *13.30* | *12.98* | *13.12* | *12.88* | *13.04* | *13.79* |
| TFe_2_O_3_ | *19.10* | *13.56* | *14.61* | *14.18* | *15.90* | *13.09* | *13.74* |
| MnO | *0.25* | *0.21* | *0.21* | *0.21* | *0.23* | *0.19* | *0.20* |
| MgO | *4.93* | *5.09* | *5.10* | *5.17* | *5.15* | *5.10* | *5.03* |
| CaO | *8.31* | *9.47* | *9.50* | *10.05* | *9.30* | *9.86* | *9.30* |
| Na_2_O | *2.44* | *2.97* | *2.65* | *2.87* | *2.63* | *3.21* | *2.80* |
| K_2_O | *0.97* | *1.06* | *1.01* | *0.84* | *1.11* | *0.60* | *1.03* |
| P_2_O_5_ | *0.24* | *0.27* | *0.27* | *0.25* | *0.25* | *0.28* | *0.27* |
| LOI | *1.94* | *1.74* | *1.78* | *1.90* | *1.66* | *2.02* | *1.92* |
| Total | *99.68* | *100.33* | *99.95* | *100.06* | *100.23* | *99.95* | *99.96* |
| FeO | *12.47* | *10.05* | *10.60* | *10.22* | *10.81* | *9.55* | *9.87* |
| Mg# | *33.67* | *42.44* | *40.68* | *41.74* | *38.90* | *43.36* | *41.84* |
| Th | 3.18 | 2.54 | 2.48 | 2.26 | 2.29 | 2.58 | 2.63 |
| U | 0.61 | 0.55 | 0.52 | 0.49 | 0.48 | 0.53 | 0.70 |
| Nb | 14.84 | 14.93 | 13.33 | 13.41 | 13.55 | 13.80 | 13.82 |
| Ta | 1.02 | 0.99 | 0.89 | 0.87 | 0.89 | 0.92 | 0.92 |
| Sr | 237.77 | 185.99 | 277.32 | 181.91 | 220.44 | 190.11 | 251.51 |
| Zr | 181.69 | 159.23 | 154.61 | 146.62 | 147.04 | 155.44 | 154.82 |
| Hf | 5.25 | 4.52 | 4.39 | 4.14 | 4.15 | 4.41 | 4.38 |
| Y | 36.87 | 33.67 | 34.11 | 31.83 | 32.07 | 33.64 | 33.38 |
| V | 808.42 | 374.17 | 396.69 | 440.35 | 514.85 | 377.24 | 359.30 |
| Cr | 50.98 | 110.99 | 104.98 | 122.94 | 106.86 | 118.58 | 134.68 |
| Co | 62.71 | 37.46 | 41.89 | 39.56 | 42.51 | 38.75 | 40.13 |
| Ni | 33.49 | 34.22 | 38.07 | 39.69 | 46.87 | 35.29 | 37.06 |
| La | 18.10 | 16.72 | 17.12 | 15.48 | 16.05 | 16.89 | 16.97 |
| Ce | 41.58 | 36.95 | 37.74 | 34.09 | 35.14 | 37.16 | 37.45 |
| Pr | 6.05 | 5.30 | 5.44 | 4.93 | 5.04 | 5.45 | 5.41 |
| Nd | 26.54 | 23.06 | 23.19 | 21.63 | 21.96 | 23.33 | 23.18 |
| Sm | 7.46 | 6.07 | 6.18 | 5.80 | 5.87 | 6.17 | 6.18 |
| Eu | 2.30 | 2.06 | 2.05 | 1.92 | 1.93 | 1.98 | 2.01 |
| Gd | 7.61 | 6.49 | 6.68 | 6.13 | 6.24 | 6.58 | 6.46 |
| Tb | 1.28 | 1.13 | 1.16 | 1.06 | 1.07 | 1.13 | 1.12 |
| Dy | 8.13 | 6.58 | 6.84 | 6.42 | 6.49 | 6.84 | 6.77 |
| Ho | 1.69 | 1.37 | 1.43 | 1.34 | 1.34 | 1.40 | 1.41 |
| Er | 4.60 | 3.65 | 3.74 | 3.46 | 3.50 | 3.69 | 3.67 |
| Tm | 0.66 | 0.52 | 0.53 | 0.50 | 0.50 | 0.53 | 0.52 |
| Yb | 4.17 | 3.30 | 3.35 | 3.17 | 3.17 | 3.36 | 3.35 |
| Lu | 0.64 | 0.49 | 0.50 | 0.47 | 0.48 | 0.51 | 0.50 |

a, the values of the reference materials; b, the standard deviation of the reference materials; c, the analyses of reference materials analysed with our samples; d, the replicate analyses of our samples.

Mg# = molar value of 100*MgO/ (FeOt + MgO); FeOt = FeO + 0.8998*Fe_2_O_3_; ferrous iron is measured by titration.
